# (*E*)-1-[4-(3-Bromo­prop­oxy)phen­yl]-2-*p*-tolyl­diazene

**DOI:** 10.1107/S1600536812021538

**Published:** 2012-05-16

**Authors:** Zhen-Xiang Yu, Bao Li

**Affiliations:** a Department of Respiratory Medicine, First Hospital, Jilin University, Changchun 130021, People’s Republic of China; b State Key Laboratory of Supramolecular Structure and Materials, Jilin University, Changchun 130012, People’s Republic of China

## Abstract

In the title mol­ecule, C_16_H_17_BrN_2_O, the benzene rings, bridged by a diazene fragment, form a dihedral angle of 6.3 (2)°. The crystal packing exhibits relatively short Br⋯Br contacts of 3.6989 (14) Å.

## Related literature

For the crystal structure of (*E*)-4-(*p*-tolydiazen­yl)phenol, see: Petek *et al.* (2006 ▶). For details of the synthesis, see: Badawi *et al.* (2006 ▶).
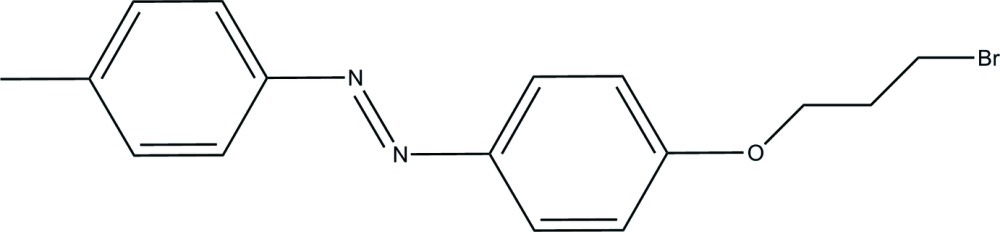


## Experimental

### 

#### Crystal data

C_16_H_17_BrN_2_O
*M* *_r_* = 333.23
Monoclinic, 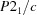

*a* = 26.530 (15) Å
*b* = 4.785 (2) Å
*c* = 11.810 (7) Å
β = 102.85 (2)°
*V* = 1461.8 (13) Å^3^
*Z* = 4
Mo *K*α radiation
μ = 2.81 mm^−1^
*T* = 293 K
0.24 × 0.23 × 0.22 mm

#### Data collection

Rigaku R-AXIS RAPID diffractometer
Absorption correction: multi-scan (*ABSCOR*; Higashi, 1995 ▶) *T*_min_ = 0.553, *T*_max_ = 0.574
13590 measured reflections
3337 independent reflections
1860 reflections with *I* > 2σ(*I*)
*R*_int_ = 0.080

#### Refinement

*R*[*F* ^2^ > 2σ(*F* ^2^)] = 0.056
*wR*(*F* ^2^) = 0.180
*S* = 1.01
3337 reflections
182 parameters
H-atom parameters constrained
Δρ_max_ = 0.40 e Å^−3^
Δρ_min_ = −0.59 e Å^−3^

### 

Data collection: *RAPID-AUTO* (Rigaku, 1998 ▶); cell refinement: *RAPID-AUTO*; data reduction: *CrystalStructure* (Rigaku/MSC, 2002 ▶); program(s) used to solve structure: *SHELXS97* (Sheldrick, 2008 ▶); program(s) used to refine structure: *SHELXL97* (Sheldrick, 2008 ▶); molecular graphics: *SHELXTL* (Sheldrick, 2008 ▶); software used to prepare material for publication: *SHELXL97*.

## Supplementary Material

Crystal structure: contains datablock(s) global, I. DOI: 10.1107/S1600536812021538/cv5298sup1.cif

Structure factors: contains datablock(s) I. DOI: 10.1107/S1600536812021538/cv5298Isup2.hkl

Supplementary material file. DOI: 10.1107/S1600536812021538/cv5298Isup3.cml

Additional supplementary materials:  crystallographic information; 3D view; checkCIF report

## Figures and Tables

**Fig. 1.**
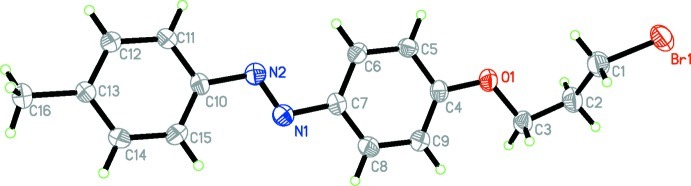
Molecular structure of (I) with displacement ellipsoids drawn at the 30% probalility level.

## supplementary crystallographic information

### Comment

The azobenzene and its derivate have been widely investigated due to the trans-cis transformations of N=N double bond caused by heating, light or other effects. Herein, we report the crystal structure of the title compound (I), which is a new derivate of azobenzene.

In (I) (Fig. 1), all bond lengths and angles are in normal ranges and comparable with those in similar structure (Petek *et al.*, 2006). The dihedral angle between the two aromatic rings is 6.3 (2)°. The crystal packing exhibits relatively short intermolecular Br···Br contacts of 3.6989 (14) Å.

### Experimental

The title compound was prepared by refluxing 4-((4-methylphenyl)azo)phenol with 1,3-dibromopropane in acetone according to the known procedure (Badawi *et al.* 2006). Single crystals suitable for X-ray diffraction were obtained by slow evaporation method at room temperature.

### Refinement

C-bound H-atoms were placed in calculated positions (C—H 0.93 to 0.97 Å) and were included in the refinement in the riding model with *U*_iso_(H) = 1.5 or 1.2 *U*_eq_(C).

### Crystal data

**Table:**

C_16_H_17_BrN_2_O	*F*(000) = 680
*M**_r_* = 333.23	*D*_x_ = 1.514 Mg m^−^^3^
Monoclinic, *P*2_1_/*c*	Mo *K*α radiation, λ = 0.71073 Å
Hall symbol: -P 2ybc	Cell parameters from 6956 reflections
*a* = 26.530 (15) Å	θ = 3.4–27.5°
*b* = 4.785 (2) Å	µ = 2.81 mm^−^^1^
*c* = 11.810 (7) Å	*T* = 293 K
β = 102.85 (2)°	Block, yellow
*V* = 1461.8 (13) Å^3^	0.24 × 0.23 × 0.22 mm
*Z* = 4	

### Data collection

**Table:**

Rigaku R-AXIS RAPID diffractometer	3337 independent reflections
Radiation source: fine-focus sealed tube	1860 reflections with *I* > 2σ(*I*)
Graphite monochromator	*R*_int_ = 0.080
ω scans	θ_max_ = 27.5°, θ_min_ = 3.2°
Absorption correction: multi-scan (*ABSCOR*; Higashi, 1995)	*h* = −34→33
*T*_min_ = 0.553, *T*_max_ = 0.574	*k* = −6→6
13590 measured reflections	*l* = −15→15

### Refinement

**Table:**

Refinement on *F*^2^	Primary atom site location: structure-invariant direct methods
Least-squares matrix: full	Secondary atom site location: difference Fourier map
*R*[*F*^2^ > 2σ(*F*^2^)] = 0.056	Hydrogen site location: inferred from neighbouring sites
*wR*(*F*^2^) = 0.180	H-atom parameters constrained
*S* = 1.01	*w* = 1/[σ^2^(*F*_o_^2^) + (0.0906*P*)^2^] where *P* = (*F*_o_^2^ + 2*F*_c_^2^)/3
3337 reflections	(Δ/σ)_max_ = 0.012
182 parameters	Δρ_max_ = 0.40 e Å^−^^3^
0 restraints	Δρ_min_ = −0.59 e Å^−^^3^

### Special details

**Table:**

Experimental. (See detailed section in the paper)
Geometry. All e.s.d.'s (except the e.s.d. in the dihedral angle between two l.s. planes) are estimated using the full covariance matrix. The cell e.s.d.'s are taken into account individually in the estimation of e.s.d.'s in distances, angles and torsion angles; correlations between e.s.d.'s in cell parameters are only used when they are defined by crystal symmetry. An approximate (isotropic) treatment of cell e.s.d.'s is used for estimating e.s.d.'s involving l.s. planes.
Refinement. Refinement of *F*^2^ against ALL reflections. The weighted *R*-factor *wR* and goodness of fit *S* are based on *F*^2^, conventional *R*-factors *R* are based on *F*, with *F* set to zero for negative *F*^2^. The threshold expression of *F*^2^ > σ(*F*^2^) is used only for calculating *R*-factors(gt) *etc*. and is not relevant to the choice of reflections for refinement. *R*-factors based on *F*^2^ are statistically about twice as large as those based on *F*, and *R*- factors based on ALL data will be even larger.

### Fractional atomic coordinates and isotropic or equivalent isotropic displacement parameters (Å^2^)

**Table:**

	*x*	*y*	*z*	*U*_iso_*/*U*_eq_	
Br1	0.03507 (2)	1.74980 (10)	0.35896 (4)	0.0780 (3)	
C1	0.07293 (17)	1.5076 (9)	0.4814 (4)	0.0666 (12)	
H1A	0.0493	1.3736	0.5031	0.080*	
H1B	0.0990	1.4048	0.4527	0.080*	
C2	0.09770 (19)	1.6700 (8)	0.5834 (4)	0.0638 (12)	
H2A	0.0716	1.7772	0.6102	0.077*	
H2B	0.1219	1.8008	0.5617	0.077*	
C3	0.12642 (16)	1.4854 (7)	0.6820 (3)	0.0555 (10)	
H3A	0.1414	1.5984	0.7493	0.067*	
H3B	0.1029	1.3514	0.7042	0.067*	
C4	0.19783 (14)	1.1643 (7)	0.7148 (3)	0.0438 (8)	
C5	0.23339 (14)	1.0199 (7)	0.6659 (3)	0.0461 (9)	
H5	0.2351	1.0542	0.5893	0.055*	
C6	0.26574 (16)	0.8279 (7)	0.7304 (3)	0.0475 (9)	
H6	0.2897	0.7334	0.6977	0.057*	
C7	0.26299 (16)	0.7723 (6)	0.8464 (4)	0.0436 (9)	
C8	0.22842 (15)	0.9224 (7)	0.8937 (3)	0.0486 (9)	
H8	0.2272	0.8924	0.9708	0.058*	
C9	0.19546 (15)	1.1169 (7)	0.8291 (3)	0.0475 (9)	
H9	0.1720	1.2144	0.8621	0.057*	
C10	0.35721 (16)	0.2505 (6)	0.9470 (3)	0.0426 (8)	
C11	0.39446 (15)	0.1260 (8)	0.8990 (3)	0.0498 (9)	
H11	0.3969	0.1742	0.8241	0.060*	
C12	0.42806 (15)	−0.0681 (7)	0.9600 (3)	0.0490 (9)	
H12	0.4531	−0.1474	0.9260	0.059*	
C13	0.42526 (14)	−0.1473 (7)	1.0710 (3)	0.0395 (8)	
C14	0.38674 (14)	−0.0307 (7)	1.1179 (3)	0.0453 (9)	
H14	0.3832	−0.0870	1.1911	0.054*	
C15	0.35311 (15)	0.1696 (7)	1.0573 (3)	0.0470 (9)	
H15	0.3279	0.2489	1.0909	0.056*	
C16	0.46205 (15)	−0.3593 (9)	1.1367 (3)	0.0514 (10)	
H16A	0.4653	−0.3327	1.2186	0.077*	
H16B	0.4953	−0.3373	1.1183	0.077*	
H16C	0.4491	−0.5438	1.1154	0.077*	
N1	0.29418 (12)	0.5728 (6)	0.9213 (3)	0.0476 (8)	
N2	0.32604 (13)	0.4536 (6)	0.8743 (3)	0.0474 (8)	
O1	0.16630 (11)	1.3435 (5)	0.6400 (2)	0.0526 (7)	

### Atomic displacement parameters (Å^2^)

**Table:**

	*U*^11^	*U*^22^	*U*^33^	*U*^12^	*U*^13^	*U*^23^
Br1	0.0757 (4)	0.0956 (5)	0.0551 (3)	−0.0036 (3)	−0.0017 (3)	0.0109 (2)
C1	0.059 (3)	0.072 (3)	0.064 (3)	0.003 (2)	0.005 (2)	0.000 (2)
C2	0.064 (3)	0.057 (2)	0.062 (3)	0.011 (2)	−0.003 (2)	−0.004 (2)
C3	0.056 (3)	0.052 (2)	0.056 (2)	0.0144 (19)	0.005 (2)	−0.0005 (18)
C4	0.038 (2)	0.0410 (17)	0.048 (2)	−0.0028 (15)	0.0019 (17)	−0.0006 (16)
C5	0.046 (2)	0.052 (2)	0.0408 (19)	−0.0021 (17)	0.0106 (17)	0.0005 (17)
C6	0.043 (2)	0.0473 (19)	0.052 (2)	0.0040 (16)	0.0107 (18)	−0.0037 (16)
C7	0.044 (2)	0.0375 (17)	0.046 (2)	−0.0043 (16)	0.0021 (17)	0.0009 (15)
C8	0.055 (2)	0.047 (2)	0.045 (2)	−0.0017 (18)	0.0115 (18)	0.0027 (16)
C9	0.045 (2)	0.0459 (18)	0.051 (2)	0.0026 (17)	0.0104 (18)	0.0006 (17)
C10	0.045 (2)	0.0383 (17)	0.0410 (19)	0.0010 (16)	0.0025 (17)	0.0022 (15)
C11	0.055 (3)	0.055 (2)	0.039 (2)	0.0079 (19)	0.0093 (18)	0.0049 (17)
C12	0.053 (2)	0.050 (2)	0.046 (2)	0.0090 (18)	0.0162 (18)	0.0032 (17)
C13	0.044 (2)	0.0343 (16)	0.0392 (19)	−0.0036 (15)	0.0063 (16)	−0.0017 (14)
C14	0.052 (2)	0.0468 (19)	0.0373 (19)	−0.0034 (17)	0.0109 (17)	−0.0010 (16)
C15	0.043 (2)	0.0472 (18)	0.052 (2)	0.0009 (16)	0.0138 (19)	−0.0076 (16)
C16	0.049 (2)	0.0476 (19)	0.055 (2)	0.0024 (18)	0.005 (2)	0.0058 (17)
N1	0.0466 (19)	0.0482 (17)	0.0462 (18)	−0.0038 (15)	0.0068 (15)	−0.0052 (14)
N2	0.0490 (19)	0.0426 (16)	0.0492 (18)	−0.0005 (14)	0.0078 (15)	−0.0032 (14)
O1	0.0480 (17)	0.0547 (14)	0.0530 (16)	0.0102 (13)	0.0070 (13)	0.0100 (13)

### Geometric parameters (Å, º)

**Table:**

Br1—C1	1.948 (4)	C7—N1	1.433 (5)
Br1—Br1^i^	3.6989 (14)	C8—C9	1.384 (5)
Br1—Br1^ii^	3.6989 (14)	C8—H8	0.9300
C1—C2	1.461 (6)	C9—H9	0.9300
C1—H1A	0.9700	C10—C11	1.379 (5)
C1—H1B	0.9700	C10—C15	1.387 (5)
C2—C3	1.524 (5)	C10—N2	1.432 (4)
C2—H2A	0.9700	C11—C12	1.375 (5)
C2—H2B	0.9700	C11—H11	0.9300
C3—O1	1.435 (4)	C12—C13	1.382 (5)
C3—H3A	0.9700	C12—H12	0.9300
C3—H3B	0.9700	C13—C14	1.383 (5)
C4—O1	1.373 (5)	C13—C16	1.499 (5)
C4—C9	1.384 (5)	C14—C15	1.394 (5)
C4—C5	1.395 (5)	C14—H14	0.9300
C5—C6	1.367 (5)	C15—H15	0.9300
C5—H5	0.9300	C16—H16A	0.9600
C6—C7	1.413 (5)	C16—H16B	0.9600
C6—H6	0.9300	C16—H16C	0.9600
C7—C8	1.378 (5)	N1—N2	1.248 (4)
			
C1—Br1—Br1^i^	103.19 (13)	C7—C8—C9	121.6 (4)
C1—Br1—Br1^ii^	176.05 (13)	C7—C8—H8	119.2
Br1^i^—Br1—Br1^ii^	80.61 (4)	C9—C8—H8	119.2
C2—C1—Br1	111.0 (3)	C4—C9—C8	119.2 (4)
C2—C1—H1A	109.4	C4—C9—H9	120.4
Br1—C1—H1A	109.4	C8—C9—H9	120.4
C2—C1—H1B	109.4	C11—C10—C15	118.6 (3)
Br1—C1—H1B	109.4	C11—C10—N2	114.7 (3)
H1A—C1—H1B	108.0	C15—C10—N2	126.7 (3)
C1—C2—C3	112.2 (3)	C12—C11—C10	121.1 (3)
C1—C2—H2A	109.2	C12—C11—H11	119.5
C3—C2—H2A	109.2	C10—C11—H11	119.5
C1—C2—H2B	109.2	C11—C12—C13	121.1 (3)
C3—C2—H2B	109.2	C11—C12—H12	119.4
H2A—C2—H2B	107.9	C13—C12—H12	119.4
O1—C3—C2	107.0 (3)	C14—C13—C12	118.0 (3)
O1—C3—H3A	110.3	C14—C13—C16	121.3 (3)
C2—C3—H3A	110.3	C12—C13—C16	120.7 (3)
O1—C3—H3B	110.3	C13—C14—C15	121.2 (3)
C2—C3—H3B	110.3	C13—C14—H14	119.4
H3A—C3—H3B	108.6	C15—C14—H14	119.4
O1—C4—C9	125.2 (3)	C10—C15—C14	119.9 (3)
O1—C4—C5	114.5 (3)	C10—C15—H15	120.0
C9—C4—C5	120.3 (3)	C14—C15—H15	120.0
C6—C5—C4	120.1 (3)	C13—C16—H16A	109.5
C6—C5—H5	120.0	C13—C16—H16B	109.5
C4—C5—H5	120.0	H16A—C16—H16B	109.5
C5—C6—C7	120.3 (3)	C13—C16—H16C	109.5
C5—C6—H6	119.8	H16A—C16—H16C	109.5
C7—C6—H6	119.8	H16B—C16—H16C	109.5
C8—C7—C6	118.5 (3)	N2—N1—C7	112.6 (3)
C8—C7—N1	116.2 (3)	N1—N2—C10	113.7 (3)
C6—C7—N1	125.2 (3)	C4—O1—C3	117.6 (3)

Symmetry codes: (i) −*x*, *y*−1/2, −*z*+1/2; (ii) −*x*, *y*+1/2, −*z*+1/2.
